# Biology and Behaviour of *Aedes aegypti* in the Human Environment: Opportunities for Vector Control of Arbovirus Transmission

**DOI:** 10.3390/v15030636

**Published:** 2023-02-27

**Authors:** Luca Facchinelli, Athanase Badolo, Philip J. McCall

**Affiliations:** 1 Department of Vector Biology, Liverpool School of Tropical Medicine, Liverpool L3 5QA, UK; 2 Laboratoire d’Entomologie Fondamentale et Appliquée, Université Joseph KI-ZERBO, Ouagadougou 03 BP 7021, Burkina Faso

**Keywords:** *Aedes aegypti*, arboviruses, domestic and peridomestic behaviour, exophagy, endophily, vector control, insecticide, indoor residual spraying, targeting

## Abstract

*Aedes aegypti* is a ubiquitous vector of arboviruses mostly in urbanised areas throughout the tropics and subtropics and a growing threat beyond. Control of *Ae. aegypti* is difficult and costly, and no vaccines are available for most of the viruses it transmits. With practical control solutions our goal, ideally suitable for delivery by householders in affected communities, we reviewed the literature on adult *Ae. aegypti* biology and behaviour, within and close to the human home, the arena where such interventions must impact. We found that knowledge was vague or important details were missing for multiple events or activities in the mosquito life cycle, such as the duration or location of the many periods when females rest between blood feeding and oviposition. The existing body of literature, though substantial, is not wholly reliable, and evidence for commonly held “facts” range from untraceable to extensive. Source references of some basic information are poor or date back more than 60 years, while other information that today is accepted widely as “fact” is not supported by evidence in the literature. Many topics, e.g., sugar feeding, resting preferences (location and duration), and blood feeding, merit being revisited in new geographical regions and ecological contexts to identify vulnerabilities for exploitation in control.

## 1. Introduction

Recognisable with the naked eye, this distinctive black and white mosquito was assigned the identity *Aedes aegypti* by Linnaeus in 1762, years before any other species were assigned to the same genus by Meigen in 1818 and long before Theobald recognised the (sub)-genus *Stegomyia*, in 1901. Over a century of taxonomy later, *Aedes* (*Stegomyia*) *aegypti* 2can be described as a polymorphic species that originated in Africa before spreading worldwide [1,2,3]. For ease of communication, in this review we oversimplify as many before us have done [2,3] and refer to *Ae. Aegypti*, which comprises at least two distinct forms, *Ae. aegypti formosus* (Walker) (*Aaf*) and *Aedes aegypti aegypti* (L.) (*Aaa*). The ancestral form *Aaf* is not known to have ever occurred outside Africa and today is found in many habitats within Africa, including both forests and urban locations [2,4]. This form is not specialized on human hosts or habitats and may play a less important role in spreading human disease [5,6,7], although some populations in West Africa are competent vectors of flaviviruses [8,9] and are found in sympatry with *Aaa* using human made containers as larval habitats [10,11]. The highly anthropophilic and therefore the most familiar form is *Aaa*, most likely differentiated from *Aaf* around 4000–6000 years ago, when the aridification of northern Africa pushed the limit of *Aaf* further south [12]. The mosquito populations left behind adapted to a life cycle within human settlements, which would have been the only sources of water in land destined to become the arid Sahel, bordering the Sahara Desert [12]. Eventually, *Aaa* spread from Africa along with the human movements, often on long journeys where conditions would have exerted further pressure to highly synanthropic behaviour [4,5,13]. *Aedes aegypti formosus* is not extensively discussed in this review, because its epidemiological role in sub-Saharan Africa is not well characterised, although the vector competence for several of its populations has been shown ([14] and references therein). Ecological studies on this subspecies in Africa lag behind the rest of the world [11] and are urgently needed if we want to reveal its role in the epidemiology of the frequent, potentially devastating, and often neglected epidemics of arboviral diseases in the African continent [15,16].

In the twenty-first century, *Aaa* has become a resilient pest of urban areas throughout the tropics and sub-tropics, as much a part of the human environment as rats, cockroaches, or houseflies. Over the past fifty years, its proliferation was enabled by an unprecedented worldwide increase in urbanisation by an ever-growing human population, and their threat to public health was elevated by the spread of many arboviruses and viral strains beyond their original ranges, a side effect of increased global travel and intercontinental trade [17]. Rising global temperatures resulting from anthropogenic climatic change have also contributed to this growth [18]. The same period of time saw the global spread of all four dengue serotypes [19], the emergence and spread of chikungunya [20], and the 2016 Zika pandemic and public health emergency [21]. Over half the world’s population are currently at risk of dengue, with 400 million cases estimated to occur annually [19]. *Aedes aegypti aegypti* remains an important urban vector of yellow fever (YF), which kills 30,000 annually with more possible if one of the urban outbreaks that still threaten Africa and Brazil [22,23] becomes a reality. The geographic expansion in the distribution of *Aaa* went beyond its expected boundaries of the tropical and sub-tropical areas of the world. Today this species also has established itself in temperate regions [24,25,26,27] and small transient populations have been discovered as far north as Germany [28] and Canada [29]. 

Responsibility for the worldwide expansion of *Aaa*-borne arboviruses into a global public health threat may have been unavoidable but it lies firmly with human society itself. Gubler 2011 [30] described an unholy trinity of urbanisation, travel and inadequate vector control, to which anthropogenic climate change has since been added [31]. Hence, as vector biologists, we must be aware that without significant changes to urbanisation trends and human movement, and a reversal in the trend of anthropogenic climatic change, controlling the densities and the spread of this vector will continue to be a serious challenge, and its elimination unachievable [31].

### Global Control Programme Hopes and Ambitions

Although the control of urban *Ae. aegypti* populations has become difficult and expensive to sustain [32,33] and references therein, it was not always so. The “*Aedes aegypti* Eradication Program” started in 1947 promoted by Brazil and endorsed by the Pan-American Health Organization (PAHO). By 1964, only seventeen years later, *Ae. aegypti* had been eliminated from 19 countries in South America [34,35], a remarkable achievement. However, the programme was never completed for a variety of reasons that can be broadly described as changes in health priorities and a consequent lack of funding, political destabilization of some Latin American countries, a loss of enthusiasm for the project, compounded by resistance to DDT in some mosquito populations [34,35,36]. After the Programme ended, *Aaa* re-colonised areas from where it had been eliminated and resumed its expansion to new territories [37,38] and the window of opportunity was lost. Although an *Aaa* eradication program was promoted in Cuba [39], and a new eradication plan was advocated in the Americas [40], insecticide resistance had arrived and the game had changed dramatically [41]. The radical changes that created modern human societies and their urban environment simultaneously favoured the spread and the increase in *Aaa* populations and created new routes for arbovirus dispersal [17,42,43]. Essentially, in recent decades, poverty and uncontrolled urbanisation together with a disregard for the basic principles of public health [43], transformed cities into ideal environments for *Ae. aegypti* [43,44], such that today, it would be difficult, if not impossible, to eliminate this mosquito from entire countries by means of the conventional techniques as was done in the last century. 

Underpinning the expansion of *Ae. aegypti* and the enormous public health challenge it has become, is the unique combination of behaviours that have enabled this mosquito to proliferate and adapt, even at the highest human densities. In this review, following the phases of the adults’ life cycle, we explore the evidence in the literature from which our perception and understanding of *Aaa* behaviour derives, with the hope of clarifying or refining current knowledge and identifying gaps with potential for improving or advancing existing control approaches [17,45].

## 2. Materials and Methods

We conducted an electronic literature review on PubMed database and Google search using *Aedes aegypti* and *Stegomyia fasciata*, associated with the following terms: biology, bionomics, physiology, distribution, behaviour, breeding/larval sites, emergence, mating, maturation, biting, host seeking, blood feeding, resting, oviposition, dispersal, control, domestic, and peridomestic. From the large body of literature available on this species, we selected the research articles and reviews in English that provided the most thorough information on any topics related to the domestic and peridomestic behaviour of *Aaa.* We used the references cited in those selected publications to trace back and search for the origins of particular information and to attempt to cross-validate the sometimes well accepted but non-evidence-based, facts. Results are reported according to the adult life and gonotrophic cycle development phases, and we discuss the main controversies and knowledge gaps identified in *Aaa* domestic and peridomestic behaviour. We have endeavoured to report objectively, presenting both the general consensus and any opposing opinions on all topics, where they existed.

## 3. Results and Discussion

### 3.1. Post-Emergence and Dispersal

Adult mosquitoes can fly within a few minutes of emergence, although development continues and maturity is not reached until 24–48 h later [46]. Adult *Aaa* are reported to rest for the first few hours to allow the exoskeleton and wings to harden [47,48]. Males have faster larval development and post-emergence, they must undergo a permanent 180° rotation of the terminalia to be able to mate [49], a process requiring 18–24 h [50]. Antennae must also mature before males can locate females through a process that requires the erection of fibrillar hairs. This character is linked to male sexual maturation [51], and it is completed 15–24 h after emergence [52]. Females are not attractive to males within several hours or the first 2.5 h post-emergence [52,53], although it has been reported that adults begin to copulate as soon as they are able to fly [48,54]. However, it has also been demonstrated that no insemination occurs before 48–72 h [55,56], i.e., young females will copulate but do not accept sperm until 2 days or older [57]. In extensive field experiments in southern Florida [58] it was shown that the age of females at insemination depended on prevailing temperatures, occurring in spring and autumn in females aged 48 h after emergence and in summer, in females aged 24 h after emergence.

Salivary glands in newly emerged females need a period of time to mature [59], which may explain why blood meals are not accepted during the first 18–24 h or 20–40 h [60,61]. Clements [62] reported that ovaries in mosquitoes continued developing after emergence at the expense of the reserves in well-nourished individuals while in undernourished individuals, sugar feeding was necessary.

The existence of a sugar feeding habit in *Aaa* is controversial. Females are reported to feed on nectar only rarely in Thailand and Puerto Rico [63,64] where they use human blood as a source of energy [64,65], although they can sugar feed to replenish energy reserves when hosts are not available [66]. This would lead to an increase in the probability for arboviral circulation [67,68], in particular during the dry season [69]. Other studies in Ecuador [70], Mexico [71] and Mali (where the presence of both *Aaa* and *Aaf* cannot be excluded) [72] report quite a different habit, with *Aaa* females frequently feeding on sugar sources in urban areas.

Several authors described the physiological changes occurring after adults emerge [46,52,53,55,56,57,59], but the information on their behaviour before insemination and blood feeding occur are not as detailed or complete. The time from adult emergence to mating/blood feeding was suggested as an opportune period allowing dispersal from the larval habitat site [73]. Together with the initial female refractoriness to insemination, this has been hypothesise as a mechanism to avoid inbreeding [73]. In a series of mark-release-recapture (MRR) experiments it was shown that adults aged 0 to 12 h post-emergence dispersed within 100 m from the release point [58]. Nelson 1986 [47] stated that adults rest a few hours on the internal walls of the container from which they emerged. Bowen 1991 [74] is cited as reporting that adults rest for 24 h after emergence [75], but we could not trace back this information, as it is not present in the cited publication [74], nor have we found any evidence for such a lengthy resting time elsewhere.

Dispersal is an aspect of *Aaa* behaviour that has potentially critical implications for population control and replacement strategies based on the release of irradiated [76], *Wolbachia* infected [77], and transgenic individuals [78]. It is also an important parameter for modelling spatiotemporal arbovirus transmission in human population and in planning of mosquito control activities [79].

The dispersal of *Aaa* has been extensively studied through mark-release-recapture MMR experiments ([80] and references therein), and genetic markers [77], reporting either limited (<100 m) or thorough (~1000 m and beyond) dispersal by female *Aaa*. It is important to point out that this species has been shown to be capable of flying long distances both in the laboratory using the flight mill (>14 km) [81] and in the field (up to 2.5 km) [82], but this probably reflects only its flight potential. Field experiments reporting long-distance flights have been performed in critical conditions with females released in a desert [82], from a boat 900 m offshore [83], or with their mouthparts sealed with glue before being liberated [84]. However, we belong to a school of thought that considers the behaviours and biological limits measured under these extreme conditions to be poorly representative of the conditions and circumstances experienced by a healthy unimpaired individual mosquito in nature. It is more realistic that *Aaa* dispersal is affected by the availability and density of houses, hosts, and larval development sites. In support of that, a study performed in semi-field conditions demonstrated that oviposition site density can influence the flight distance of this species [85].

There is agreement between multiple authors [46,47,52,53,55,56,57], that after emergence from pupae, adult mosquitoes need to mature before insemination occurs and the gonotrophic cycle starts, but little to no data or evidence exist that describe those behaviours that are common within this timeframe. Many unanswered questions remain: where are females during this period of time (24–72 h), in nature? Do they feed on sugar sources or blood for energetic purposes? Do they disperse and how far? Where do they rest and for how long? 

### 3.2. Behaviour and Biology: Mating

The order of precedence of mating and blood feeding differs among mosquito species [86] and *Aaa* females are reported to take the first blood meal both before and after insemination [73,87]. Although males swarm near dusk and dawn if a host is not available [88], mating usually occurs near the host, to where both males and females are attracted [73]. As a consequence, mating and the first blood feeding can occur almost simultaneously [47]. Males perform nuptial flights near the host with the typical horizontal figure-eight pattern waiting for host-seeking females [73]. During courtship, males and females interact acoustically by matching the pitch of their flight tones [89]. Aggregation of both sexes for mating purposes seems to be also mediated by a male-produced pheromone [88,90], and epicuticular hydrocarbons have been hypothesised to play a role in female sexual receptivity [91]. The mosquito auditory organs, the antennae, play a crucial role in mating. Cator et al. 2009 [89], demonstrated that during courtship, male and female *Aaa* shift their flight tones to match at a shared frequency much higher in pitch than the fundamental tones of the two sexes. Further studies [92,93] have exploited this finding, utilising the harmonic convergence behaviour as a predictor for male fitness, since male mating performance represents a crucial prerequisite for the success of genetic control approaches. Females are tendentially monogamous [94], although different levels of polyandry have been documented both in large field cages and in open field [95,96,97]. Male accessory gland content induces monogamy in inseminated females [98,99,100], and is also responsible. for increasing their longevity [101]. Mating is reported to occur both inside and outside houses [87,102], with a bimodal pattern similarly to what is described for the female daily biting activity [87,103,104,105]. 

Fuchs & Kang [106] reported that females rest for 12 h after mating [75,107]. This would be a promising behavioural event to target, if confirmed, but we did not find any mention of it in that paper [106], and we have not found any other report of its existence based on evidence in the literature.

In different mosquito vector species, males return repeatedly to congregate in large swarms in defined mating arenas close to larval rearing sites or inside/nearby villages [108,109,110]. This behaviour has been successfully targeted with insecticides to control *Anopheles gambiae* populations where mating occurs at the breeding site [111], something that would not be repeatable with *Aaa*, since most of the mating events occur in close proximity to the host in domestic and peri domestic environment [73]. However, elucidating the molecular and physiological mechanisms controlling mating behaviour in *Aaa* is of paramount importance for *Wolbachia* [112] and genetic-based control, techniques [113] that rely on successful mating of released individuals. Mating competitivity and reproductive fitness [114] of mass-reared males and, more generally, of individuals bearing a desired trait, can be assessed and reliably employed to parameterise mathematical models to effectively predict the output of field releases when a full understanding of the natural mosquito mating system is achieved [115].

### 3.3. Behaviour and Biology: Host Seeking, Blood and Sugar Feeding

**Figure 1:**
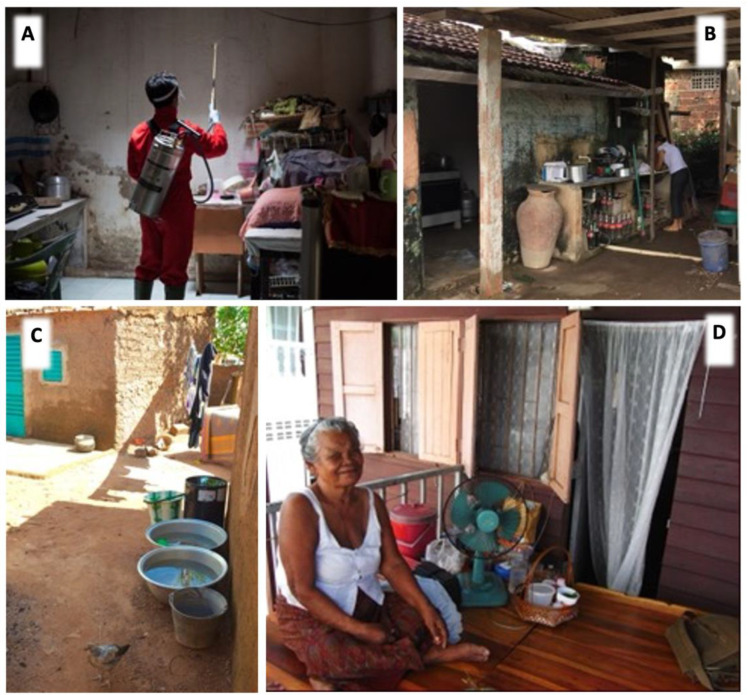
Vector preferences for host-seeking and resting behaviour influence vector control interventions as does the community’s response to them. (**A**) Standard IRS involves the application of aqueous insecticide residues on indoor walls and ceiling and is most effective for vector control where endophily is the predominant behaviour; in a busy room such as this, where are the preferential resting sites? What could guide us in efforts to identify the spots preferred by *Ae. aegypti*, e.g., surface colour or texture, light intensity, flight paths, degree of cover, etc. to reliably identify them as target sites? (**B**) Where is, or what would be defined as indoor or outdoor in this dwelling in Recife, Brazil? (**C**) Ouagadougou Burkina Faso. Here, in a relatively arid region, the *Ae. aegypti* population is highly exophilic, but with a disproportionately high number of bloodfed females caught inside houses (see Badolo et al. 2022). This is in contrast to (**D**) Chon Buri in Thailand, where despite the high humidity, most biting occurs while humans are indoors. However, it is very hot indoors and people prefer to spend leisure time outdoors, negating the impact of the insecticide-treated curtains (visible in the doorway and windows behind), which form a barrier to house entry but contribute little to vector control as a result. (Figure 1A © Chris Barrett; Figure 1B–D © PJ McCall).

Female *Aaa* display relentless host seeking behaviour [116] throughout their lives. Exhaled by the host, carbon dioxide activates host-seeking and reduces the threshold for the detection of skin odorants [117], which play an important role in host approach, together with vision, host body temperature, and humidity [118]. McDonald (1956) [119] reported “there is some confusion” on the preferred biting time, with a general agreement that females of this species bite principally by day but with several studies reporting some night activity indoors (Ref. [119] and references therein). The propensity for domestic populations of *Aaa* to access houses in east Africa searching for a blood meal has been shown to be a heritable trait that differentiates them from the sylvan populations in the same area [120]. Day biting vectors exploit the sit-and-wait strategy [121], with *Aaa* being reported numerous times over many decades to bite indoors, a characteristic of this species [122,123] at least in Asia and the Americas, where most studies have been performed. In a recent study in Burkina Faso, West Africa, adult collections indicated a highly exophilic vector population but with a relatively high proportion of blood fed females caught inside houses, suggesting endophagy [124]. Outdoor biting activity has also been described in some human bait studies [125,126,127], and in Southern Mexico, it was reported to be more common than indoor biting [128]. The use of outdoor traps baited to attract host-seeking females [125,129,130] has shown in several populations that exophagic activity in *Ae. aegypti* can constitute a significant proportion of total biting. The location as well as the duration of the blood feeding and resting stages of the gonotrophic cycle are important elements of most vector’s biology, as they can influence or even determine the impact of any intervention (see Figure 1). Reliable information on these behaviours in the vector population, and of the behaviour of the human population is essential before selection and implementation of any control method.

Similar to other species in the subgenus *Stegomyia*, *Aedes aegypti aegypti* typically bleed feeds multiple times within a single gonotrophic cycle [131,132]. This is regulated by the amount of blood ingested and has important consequences on host-seeking. Abdominal distension following large blood meals immediately leads to a short-term inhibition of host seeking [133,134], and a second long-term inhibition is mediated by a neuropeptide produced during oogenesis, and occurs 30 h after a large blood meal is ingested [135,136]. If females blood feed to repletion, the two mechanisms overlap and host seeking is inhibited until eggs are laid [135]. This regulation mechanism is also affected by *Aaa* females’ nutritional status. Females with limited to no access to carbohydrate sources will employ the blood meal for energetic purposes with consequent short-term inhibition of host seeking only [137] and with the increase of multiple feeding. Additionally, females deprived of carbohydrates but able to develop eggs will be more likely to suppress the long-term host-seeking inhibition mechanism than well-nourished ones [137]. Male accessory gland products affect this behaviour as well, with mated females showing a stronger host-seeking inhibition after a large blood meal with respect to unmated ones [138,139]. Host movements or defensive behaviour may cause females to take a partial blood meal, which is not sufficient to inhibit long-term host seeking, and multiple feedings within a single gonotrophic cycle are more likely to occur [132,133]. The propensity for females to feed frequently on humans for both energetic and reproductive needs serves to increase their fitness if sugar resources are not used [65] with obvious consequences for arboviral transmission [60,140].

Exploiting sugar vs. blood sources for replenishing energy reserves and the preference to take blood meals indoors vs. outdoors have direct implications in the application/development of effective strategies to control this species. For example, attractive toxic sugar baits (ATSBs) have been developed to target both male and female mosquitoes [141], and specific devices have been employed to target *Aaa* [72,142]. Attractive toxic solutions have also been sprayed on vegetation in semi-field tests [143] or field trials [72] and full large-scale field trials are needed to assess the effect of these techniques in controlling *Aaa* populations. Further research is needed to determine if geographic, environmental, and genetic variability or phenotypic plasticity may play a role in explaining the seemingly contradictory data available on these topics so far [63,64,70,71,72,125,126,127], potentially opening the way to tailored vector control strategies.

### 3.4. Behaviour and Biology: Resting and Oviposition

Once a sufficient amount of ingested blood has triggered both the inhibition of host-seeking behaviour and initiation of the gonotrophic cycle, engorged females find a sheltered place to rest and develop eggs [60,144]. A high proportion of resting *Aaa* females collected indoors are blood fed or gravid [119,124,145], which suggests a tendency to digest the blood meal and mature the eggs inside houses.

Blood fed, gravid, and unfed females are attracted to non-reflective dark surfaces [146,147] and will remain at rest on dark clothing [48,144,148], on clothing, bed covers, furniture, doors, walls, ceiling [119], in darkened areas of the rooms [149], and on both exposed and unexposed surfaces [150] in close proximity to their larval development site. Variously, they are reported to have no marked preference for any particular height [119] or prefer resting below 1.5 m height [145,150,151], but there is common agreement on bedrooms as the place in the house where the majority of individuals are collected [47,75,145,150]. 

Resting behaviour occurring in proximity of water holding containers [149], and the high number of individuals (both gravid and unfed) often collected outdoors by means of sticky ovitraps [152,153,154], suggest that exophilic resting activity of *Aaa* is not negligible. Similar to outdoor resting, it too might be the preference of a significant number of mosquitoes, as found by Perich et al. 2000 [151] who collected ~20% of resting females outdoors during a survey in Panama. Exophily may also be common in populations in sub-Saharan Africa [124].

The resting behaviour of *Aaa* can be exploited using indoor residual spray (IRS), where internal walls of habitations are sprayed on a regular basis [75,145,149,155,156]. This technique is attractive for targeting the endophilic behaviour of *Aaa*, which is known to have a sedentary life style [63,65,75], and a comprehensive practical manual to employ IRS for fighting *Aaa* in urban areas has been recently released by PAHO [157]. The manual is based on resting behaviour studies [75,145,155,158,159] and promotes a modified IRS approach called Targeted Indoor Residual Spraying (TIRS) or IRS for urban *Aedes* control (IRS-*Aedes*), which consists of applying residual insecticides only onto the lower parts of the walls and furniture where it is reported these mosquitoes preferentially rest (below 1.5 m). While this approach generates some saving in the high expenses driven by personnel and insecticides [32], it does not cope with the challenge of delivering *Aaa* control in densely populated urban environments. Moreover, although the amount of insecticide employed is greatly reduced compared to standard IRS, TIRS still requires skilled operators and specialised equipment, a feature that restricts it to vertical or centralised control programmes [160]. Facchinelli et al. 2023 recently showed in an experimental furnished room in Brazil that it is possible to further reduce the surface treated with residual insecticides to ~12% of total interior wall surface area and still deliver high mortality in a 24 h timeframe by exploiting mosquito vision. If confirmed in real houses this approach would enable householders to treat their own homes, providing indoor protection from *Ae. aegypti* bites while greatly reducing the quantity and spread of insecticide residues in the domestic environment even in high-rise high-density areas [161].

Targeting specific *Aaa* behaviours using insecticide treated materials (ITM), volatile pyrethroids emanators, and insecticides applied on limited surfaces where *Aaa* would preferentially rest, have been proposed to further decrease the amount of adulticides used in domestic and peri domestic environment [141,148,162,163,164,165,166,167]. These approaches have the potential to overcome the limitation of delivering control in high-density urban contexts using simple, possibly safer, and affordable home vector control products, promoted via education and public awareness programmes. Purchased by householders or distributed for free during arbovirus outbreaks in affected locations, these could be complementary to vertical programs permitting effective community-level control [161]. The use and effectiveness of such approaches, also in combination [168], could be considerably improved by elucidating the domestic and peridomestic *Aaa* adult behaviour in different geographic and environmental settings. 

*Aedes aegypti aegypti* gravid females lay eggs in natural and artificial water holding containers of a wide range of sizes and materials [144,169], both inside dwellings and in the peri domestic environment. Gravid females distribute eggs from a single gonotrophic cycle in several containers (skip oviposition) [170,171,172,173,174], although the latter is not always confirmed [175]. Early studies described this species developing in clean water [144]. There is a general consensus on the oviposition behaviour of *Aaa*, although in some locations, variations are reported. The first evidences of septic tanks used as larval development sites come from Malaysia [176], Nigeria [177] and Colombia [178] where, in the city of Cali they represented the most productive *Aaa* larval habitat. These findings have been subsequently confirmed in other geographical areas [179,180,181]. Interestingly, larval habitat differences (rock pools vs. domestic containers), consistent with genetic differentiation indicated *Aaa* in Anguilla island “do not constitute a single panmictic population, but there are no large consistent differences to parallel the East African sylvan-domestic dichotomy” [182]. The two populations of Anguilla were also significantly differentiated regarding development time and insecticide resistance [183]. These differences confirm that the species is highly adaptable and, as stated by Tabachnick and Powell [2], *Aaa* “maintains significant genetic variation for different life history traits and […] breeding in human-generated containers is not a fixed trait outside of Africa”.

An oviposition pheromone, heneicosane attracts gravid females and has been identified in both larval conditioned water and larval cuticle extracts [184]. Yet, *Ae. aegypti* is a species where the offspring develop in temporary water in small potentially highly competitive environments, and gravid females have been reported to sample the water before laying any eggs. Thus, *Ae. aegypti* have been reported to avoid waters holding starved larvae [185] while Chadee et al. 1990 [186] reported that, when given a choice between their own and those of conspecifics, female *Ae. Aegypti* preferentially avoided laying eggs in the presence of their own eggs. However, Torres-Estrada et al. 2001 [187] found that *Ae. aegypti* females preferred to lay eggs in water that currently or previously contained the predatory copepod *Mesocyclops longisetus*, (possibly in response to copepod-derived terpenes in the water). Studies of this type continue to be published but the promise of the use of semiochemicals as baits for attractant traps or as biting repellents has yet to be realised [188]. Geosmin, “the smell of earth after rain” is a sesquiterpenoid associated with streptomyces bacteria that mediates oviposition by *Aedes aegypti* where it is decoded by the olfactory system in a precise manner not dissimilar from that interpreting human host cues [189].

### 3.5. Modulation of behaviour through Memory and Learning

The semiochemical roles of various cues become even more complex with the knowledge that their influence on individual mosquitoes can vary depending on prior experience of the individual mosquito. Following the demonstration of learning and memory in *Culex quinquefasciatus* [190], Kaur et al. 2003 [191] used a similar design approach to show that *Aedes aegypti* gravid females mosquitoes would oviposit in water containing normally repellent chemicals if they had been reared with that repellent. The induced preference for repellent was not inherited. Vinauger et al. (2014) used a Pavlovian conditioning paradigm to show that *Aaa* could be classically conditioned [192]. They trained *Aaa* to associate an odorant conditioned stimulus (CS) with a blood-reinforced thermal stimulus (unconditioned stimulus; US); mosquitoes could learn the association between L-lactic acid and the US and retain the association as a memory for at least 24 h. A possible role for and potential importance of learning in numerous key behaviours of vectors have been considered or explored and include host fidelity, breeding site selection, mating behaviour, and dispersal [192,193,194]. While memory could exert a significant influence on vectorial capacity, such an impact has not yet been shown in *Aaa* or any other mosquito.

### 3.6. Recent Advances in Sensory Biology and Mechanisms of Host Discrimination

*Aedes aegypti* has become the laboratory model organism of choice for studies into the sensory biology of mosquitoes, including fundamental work on the perception of human vs animal host cues in the anthropophilic *Aaa* form. Some key published work is described here.

DeGennaro et al. 2013 [195] showed that mutant *Ae. aegypti* lacking functional obligate Orco co-receptors did not respond to human odours unless CO_2_ was also present. The odorant receptor was crucial for discriminating human from nonhuman hosts. They also found that mutant females were attracted to human hosts even in the presence of DEET, but were repelled on contact, indicating that olfactory and contact-mediated effects of DEET are mechanistically distinct. Subsequently, Raji et al. 2019 [196] showed that ionotropic receptors were responsible for a large proportion of human attraction that was not dependent on Orco. McBride et al. 2014 [197] reported how a preference for humans in *Aaa* was linked with the olfactory receptor *Or4*, which is more highly expressed and more sensitive than in *Aaf*. A recent work has identified one glomerulus in particular in the brain of *Ae. aegypti* that is strongly activated by human odour but barely responds to animal odour [198]. This human-sensitive glomerulus is selectively tuned to two aldehydes, decanal and undecanal, which derive from uniquely human skin lipids and comprise a substantial component of human odour. The recent study on detection of Geosmin and its role as an oviposition attractant [189] revealed the existence of a similar system in *Ae aegypti* for processing information pertaining to oviposition.

Herre et al. 2022 [199] reported that the mosquito receptors involved in detecting host odours are typically detected by neurons that co-express multiple chemosensory receptors directly affecting mosquito behaviour and challenging the canonical one-receptor-to-one-neuron organization model.

Finally, looking to tie it all together, van Breugel et al. 2015 [200] investigated interactions between olfaction and vision. They found that, although olfactory, visual, and thermal cues trigger independent host-seeking behavioural modules, the perception of CO_2_ triggers a strong attraction to visual features, while thermal target responses operate independently of CO_2_.

In addition to their contribution to the growing body of work elucidating the mechanisms of host discrimination, this research offers the potential to identify with considerable accuracy, molecules that are central to attraction and repulsion in *Ae. aegypti* and other hematophagous arthropods [201,202,203]. 

### 3.7. Which Phenotype of Aedes Aegypti am I Working With? A Note of Caution

The pantropical *Aaa* is back in west Africa, where it and the original form coexist in some locations [3], indicating the complex scenario in Africa. There is a site in the Rabai District of Kenya where sympatric populations of *Aaa* and the ancestral *Aaf* forms introgress freely in urban areas yet remain genetically distinct in rural/forest settings [3,197]. This fortuitous location has been invaluable for a wide range of studies [120,204,205] and hence is quite widely known and sometimes perhaps considered representative of *Aedes aegypti* in sub-Saharan Africa as a whole. However, the ease with which the forms are separable here is rare and not in any way typical of Africa. The complexity is increased by the reintroductions of *Aaa* in the African continent [206] and by the presence of domestic populations in West Africa [4,5]. The real picture is one of complexity and variation, as many of the useful morphological characters are rarely exclusive to one form and differentiation or determining “which form is present or predominant” at any particular study site, is far from straightforward.

## 4. Conclusions

From this review of a considerable body of research in the published literature, it is clear that there are many *Aaa* behaviours for which little is known, including some that could offer routes to more effective control (Table 1). Greater insight into resting behaviour—where?, why?, for how long?—at any stage in the life cycle has potential if it identifies a surface where residual insecticide deposits can be deployed to target the mosquito safely and accurately.

Further insight is still needed into the relative preference for indoor/outdoor resting and blood feeding and whether or not endophagy can still be considered an exclusive or dominant behaviour in this species [207,208]. Similarly, the levels of anthropophagy/zoophagy are worth investigating, particularly in different populations with known or suspected *Aaf* presence.

Sugar feeding has very high potential for use in control. The importance of a sugar feeding habit, which is variously reported in its use by *Aaa* [63,64,66,70,71], should be determined in multiple geographic areas. Oviposition site selection behaviour may be far broader than thought: several reports exist of *Aaa* colonising septic tanks [176,177,178,179,180,181], but it is not clear if they only describe local and marginal situations or additional studies could confirm whether these organically rich water sources represent an important production site worldwide or only in certain environments/geographic areas.

It might be too much to expect an entirely novel control method from the studies we are proposing here, but the refinement of existing practices following new knowledge of the vector’s behaviour can be just as beneficial. The resting preferences of *Aaa* are a crucial aspect of one behaviour targeted by IRS, and studies performed in the last decade showed that the majority of females prefer to rest on walls below 1.5 m. This led to the successful use of Targeted Indoor Residual Spraying (TIRS) [159,209], making it possible to decrease the amount of insecticide sprayed indoors, dramatically reducing the treatment time per house with no loss in efficacy compared to standard IRS. Still, we suspected that with deeper knowledge of the resting preferences of this mosquito, it might be possible to improve indoor control further by reducing the instruction on where to treat to a very simple message (Facchinelli et al., unpublished). A demonstration that only a limited area of lower wall surface requires insecticide treatment to kill as many *Ae. aegypti* as other iRS methods means that it would be possible for the home to be protected by the householder without reliance on the equipment or skills that limit vertical control programmes. Though shown only in experimental housing at present, it will be extended to the field in the near future. We are now investigating how *Aaa* interacts with the house structure including entry/exit routes and how those habits are affected by environmental conditions or particular physical characteristics.

Despite many recent advances developing innovative techniques to decrease the densities of this vector species or to alter it in some way to render it refractory to arbovirus infections [113,168,210,211], it is certain that the desire and the need to protect one’s home and family from arboviral infections will not diminish and that the contribution of individual households through community-based environmental management, source reduction, the use of larvicides, or residual insecticides brings with it the real prospect of sustainable control [168]. Indeed, many of the control approaches listed previously by Achee et al. (2015) [168] would benefit from filling the knowledge gaps in *Ae. aegypti* domestic and peridomestic behaviour and investigating their importance in geographically different populations, as highlighted in this review.

## Figures and Tables

**Table 1:** Table showing topics for which little is known, or where there is disagreement among experts or where this review’s authors consider the topic likely to be a rich area for exploration. The numbers in the right column refer to the number of the appropriate article in the reference list of this review.

Behaviour/Activity	Knowledge Gap	What is Missing?/ What’s to Investigate	Potential Use in Control	References
post emergence	24 h resting not confirmed	behaviour unclear prior to mating/blood feeding	residual insecticides	[67,68]
sugar feeding	contradictory results	possible geographic and/or seasonal and/or population variability	ATSBs	[59,60,61,62,63,64,65]
mating	Control mechanisms unclear	molecular and physiological basis regulating mating	improve the use of SIT, Wolbachia, transgenic mosquitoes	[98]
post-mating	12 h resting not confirmed	lack of data	optimize use of indoor vs. outdoor control to	[68,89,90]
endophagy	mainly described as indoor feeders but many studies report exophagic behaviour	to establish the degree of outdoor biting according to geographic and/or seasonal and/or population variability	optimize use of indoor vs. outdoor control tools	[105,106,107,108,109,110,111,112,113]
endophily	detailed indoor resting behaviour	indoor preferred resting surfaces according to house structure and/or geographic and/or seasonal and/or population variability	Enables householders’ self-protection	[144]
oviposition	Site location and selection entirely non-random; Role of vision or olfaction; though container breeders, unexpected larval sites exist	Pheromone role (attractant or repulsion), other semiochemicals; assess contribution of alternative sites (e.g., septic tanks) to *Ae. aegypti* population densities	Larval control	[154,155,156,157,158,159]

## Data Availability

Not applicable.
